# Stroke and pump thrombosis following left ventricular assist device implantation: The impact of the implantation technique

**DOI:** 10.3389/fcvm.2023.974527

**Published:** 2023-05-11

**Authors:** Michal Nozdrzykowski, Jessica-Marie Bauer, Uwe Schulz, Khalil Jawad, Christian Bireta, Sandra Eifert, Marcus Sandri, Joanna Jozwiak-Nozdrzykowska, Michael A. Borger, Diyar Saeed

**Affiliations:** ^1^ University Department of Cardiac Surgery, Leipzig Heart Center, Leipzig, Germany; ^2^ Department of Cardiology, Leipzig Heart Center, Leipzig, Germany

**Keywords:** left ventricular assist device, stroke, pump thrombosis, right ventricular assist device (RVAD), less-invasive surgery

## Abstract

**Objectives:**

Several studies have shown the potential advantage of less-invasive surgery (LIS) for left ventricular assist device (LVAD) implantation. This study aims to determine the impact of LIS on stroke and pump thrombosis events after LVAD implantation.

**Methods:**

Between January 2015 and March 2021, 335 consecutive patients underwent LVAD implantation using either conventional sternotomy (CS) or the LIS technique. Patient characteristics was prospectively collected. All patients were followed up until October 2021. Logistic multivariate regression and propensity-matched analyses were performed to account for confounding factors.

**Results:**

A total of 242 patients (*F* = 32; 13.0%) underwent LVAD implantation with CS and 93 patients (*F* = 8; 8.6%) with the LIS approach. Propensity matching generated two groups, including 98 patients in the CS group and 67 in the LIS group. Intensive care unit stay for the LIS group patients was significantly shorter than that for the CS group patients [2 (IQR: 2–5) days vs. 4 (IQR: 2–12) days, *p* < 0.01]. There were no significant differences in the incidence of stroke events (14% in CS vs. 16% in the LIS group; *p* = 0.6) or in pump thrombosis (6.1% in CS vs. 7.5% in the LIS group; *p* = 0.8) between the groups. The hospital mortality rate in the matched cohort was significantly lower in the LIS group (7.5% vs. 19%; *p* = 0.03). However, the 1-year mortality rate showed no significant difference between both groups (24.5% in CS and 17.9% in LIS; *p* = 0.35).

**Conclusions:**

The LIS approach for LVAD implantation is a safe procedure with potential advantage in the early postoperative period. However, the LIS approach remains comparable to the sternotomy approach in terms of postoperative stroke, pump thrombosis, and outcome.

## Introduction

The application of mechanical circulatory support (MCS) in patients with advanced heart failure (AHF) has increased in Europe recently because of a lack of donor organs (1). Further, MCS therapy is considered a valuable option as a destination therapy or bridge-to-transplant for patients who are currently not candidates for heart transplantation.

However, the major surgical challenge lies in the comorbidities of these patients and previous cardiac surgery operations. Conventional sternotomy (CS) using a cardiopulmonary bypass (CPB) machine is considered a standard approach for left ventricular assist device (LVAD) implantation. To reduce the invasiveness of the LVAD implantation, many centers started using less-invasive surgery (LIS) approaches. A series of studies have shown that the LIS approach for LVAD implantation results in fewer hemodialysis treatments, lower rates of right ventricular (RV) failure, fewer blood transfusions, and significantly shorter intensive care unit (ICU) and hospital stays (2–5).

Some clinicians opine that the minimally invasive approaches may be associated with a higher rate of pump thrombosis and stroke (6). This may be related to the difficulties encountered in the deairing procedure and the inability to adequately visualize the left ventricle in LIS patients. To our knowledge, there are no representative studies looking specifically at the differences in thromboembolic complications in patients undergoing LIS and CS. The main aim of this study is to investigate the impact of the VAD implantation technique on stroke and pump thrombosis events following LVAD implantation.

## Materials and methods

### Ethical statement

Written informed consent for data collection is available for all patients included in the study.

### Study population

This was a retrospective review of prospectively collected data maintained in our institutional LVAD database. All subjects in this study had to meet INTERMACS (The Interagency Registry for Mechanically Assisted Circulatory Support) eligibility criteria. The conditions of the patients were discussed in detail with the heart failure team. Included patients were implanted during the period from January 2015 through March 2021. To prove the effectiveness of the surgical technique, the patients were grouped on the basis of the surgical approach, conventional full median sternotomy (CS cohort), or the less-invasive approach (LIS cohort). The decision to proceed with sternotomy or the LIS approach was based on the operating surgeon's discretion. Both surgical techniques were used for the duration of the study; however, the sternotomy technique was used more commonly in the early years. However, after the implementation of the new procedure (LIS) at our center, it is being exclusively used unless concomitant surgery is required. The use percentage of the LIS approach has been >70% of the cases starting from 2018. Indications and perioperative management of patients with AHF showed consistent results throughout the recruitment period. No changes were made regarding anticoagulation management of LVAD patients over the course of the study period. The ventricular assist devices implanted were HeartMate II®, HeartMate III (Thoratec Corporation, Pleasanton, CA, United States), and HeartWare® (HeartWare, Incorporated, Framingham, MA, United States).

### Study variables, definitions, and outcome measures

Information related to patient demographics, comorbidities, interventions before LVAD implantation, laboratory parameters, and hemodynamic measurements was collected for all patients. Intraoperative data such as CPB time, total procedural time, and concomitant procedures were analyzed. The primary outcome was freedom from stroke and/or pump thrombosis. The secondary outcome was survival till discharge and during follow-up.

Stroke was defined according to the INTERMACS Protocol: any new, symptomatic, clinically documented neurologic dysfunction persisting beyond 24 h that is also associated with radiographic evidence of a cerebrovascular insult corresponding to the deficit. The treatment strategy for stroke was devised according to the directive of the guidelines and in consultation with a neurologist, neuroradiologist, and interventional radiologist. In patients who had undergone an LVAD implantation in the past 14 days, the indication for the intravenous application of alteplase (Actilyse® Boehringer Ingelheim Pharma GmbH&Co. KG, Ingelheim, Germany) was carefully considered and the potential increased risk of surgical-site hemorrhage was weighed against the anticipated benefits of reduced stroke-related neurological deficits. In select acute stroke patients who had a large vessel occlusion, mechanical thrombectomy was considered. Pump thrombosis was determined on the basis of clinical, biochemical, or hemodynamic findings or on the basis of device inspection or incontrovertible evidence of radiologic studies or in the absence of appropriate Doppler flow signals that confirm the presence of thrombus within the device or its conduits. The first case is referred to as “suspected” and the second one as “confirmed.” All pump thrombosis events (suspected or confirmed) were judged and acted upon according to the definition outlined in Supplementary Appendix. The treatment of pump thrombosis depended on the diagnosis of the type of blood obstruction based on clinical status, hemodynamic values, echocardiographic evaluation, level of hemolysis, and end-organ function. Thrombolysis was performed with alteplase infusion consisting of a bolus of 10 mg, followed by a bolus of 20–40 mg over 20 min, and an infusion of 1 mg/h over 24 h. Hemolysis parameters were monitored daily, such as lactate dehydrogenase, plasma-free hemoglobin, haptoglobin, total bilirubin, and hemoglobinuria. Surgical treatment option included surgical pump exchange. All explanted pumps were disassembled and visually inspected for thrombus formation. In the case of outflow graft thrombosis, the graft was stented. Stenting was performed under angiographic monitoring. In the case of recurrent pump thrombosis, the indication for urgent transplantation, according to Eurotransplant Heart Transplantation guidelines, was given. Postoperative complications were recorded according to INTERMACS definitions, including RV failure (RVF). Severe RVF was denoted by the use of a right ventricular assist device or postoperative inotropes for longer than 14 days.

### Operative techniques

All LVAD implantations were performed at a single institution by two experienced surgeons, who performed both CS and LIS techniques. The LIS procedure was introduced at our institution in 2016, and since October 2018, it has been carried out in almost all patients, except for those requiring concomitant procedures (e.g., aortic valve replacement, atrial septum defect reconstruction, etc.). The switch from conventional sternotomy to a less-invasive approach was associated with hiring a new program director at our institution.

For the LIS approach, the patient was positioned with a slight elevation of the left chest. The location of the LV apex was identified through transthoracic echocardiography and marked on the patient's skin in order to perform a minimized incision for thoracotomy. Before starting the operation, a venous guide wire was placed in the femoral vein using ultrasound guidance. In the case of reoperation, an arterial guide wire was also placed in the femoral artery. At this stage, 2000IE of heparin was administered and the femoral vein was cannulated percutaneously using the Seldinger technique under transesophageal guidance. Subsequently, surgical access was made using a partial J-shaped sternotomy in the 3rd intercostal space (ICS). Then, the aorta was cannulated for CPB and a needle vent was inserted in the ascending aorta for deairing. Venous cannulation for CPB was done *via* the previously inserted percutaneous venous cannula. In the next step, anterolateral thoracotomy at the previously marked site was performed. The pericardium was opened and the insertion site of the LVAD was localized by echocardiographic assessment. The sewing ring was then secured with interrupted pledgetted sutures. Thereafter, full-dose heparin was administered *as per* standard protocol and the CPB procedure started. The majority of patients underwent operation with a CPB machine. The apex was incised within the sewing ring using a coring knife. The device was inserted into the ventricle and fixed. The driveline was tunneled using the C-Technique (7). The outflow graft was tunneled within the pericardium and anastomosed end-to-side to the ascending aorta after a partial clamping of the ascending aorta. Deairing was performed through the outflow graft and ascending aorta. The CS consisted of a pump and outflow graft insertion *via* a median sternotomy. In both approaches, a complete coverage of the pump using a polytetrafluoroethylene membrane and closure of the pericardium over the pump were achieved. Moreover, the pericardium over the ascending aorta was also closed. In this way, the dilatation of the right ventricle could be reduced, thus facilitating easier performance of later reoperations.

Notably, only a few patients underwent off-pump implantation in this series. The off-pump series was performed predominately with the HVAD pump. None of the HM 3 patients were implanted using the off-pump approach. Both cohorts were treated postoperatively in the cardiovascular ICU by a team of intensive care specialists by administering the same postoperative goal-directed therapy.

### Statistical methods

Continuous variables are presented as a median with an interquartile range or as a mean with standard deviation, depending on the distribution. Categorical variables are presented as counts and percentages. Differences for continuous variables were determined by using the *T*-test or Mann–Whitney *U*-test and for categorical data with the Chi-square test or Fisher's exact test, and they were found to be appropriate. Patients operated using the CS approach were compared with those in whom the LIS approach was used. Because these two patient groups differed in terms of baseline parameters, a propensity score analysis was computed. The following variables were included in the propensity score match on the basis of the distribution in the groups and clinical expertise of the research team: patient age, sex, hypertension, intubation, previous cardiac surgery, whether the patient was on extracorporeal membrane oxygenation (ECMO) preoperatively, whether LVAD implantation was performed off-pump, VAD model (HeartWare, HM3), and INTERMACS profiles. First, the balance of the variables between both surgery groups was assessed and visualized using standard mean differences and absolute standard differences. Subsequently, propensity scores were generated using multivariable logistic regression analysis, and the matching of patients in the CS group with those in the LIS group was done in R with the MatchIt package using the nearest neighbor matching with a caliper distance of 0.2 standard deviation and ratio of 2, resulting in 98 CS patients matched with 67 LIS patients. For all analyses, two-tailed *p*-values <0.05 were considered statistically significant. Analyses were performed using R4.1.

## Results

### Study population and preoperative characteristics

Between January 2015 and March 2021, a total of 335 consecutive patients underwent LVAD implantation for advanced heart failure at our center; 242 implantations were performed through CS and 93 by LIS. The LIS approach was found feasible in all patients, and none of the patients were switched from LIS to CS. The median age of the LIS group was 63 years (IQR 22–66) vs. 61 years (IQR 53–66) in the CS group (*p* = 0.4). There was also no significant differences in body mass index, sex, history of diabetes, peripheral arterial disease, chronic kidney disease, history of hypertension, and type of cardiomyopathy (Table 1). The CS group consisted of 167 HM3, 14 HM2, and 61 HVADs compared with 53 HM3, 1 HM2, and 39 HVADs in the LIS group (*p* = 0.004). A total of 67 patients (28%) in the CS group had a history of cardiac surgeries vs. 13 patients (14%) in the LIS group (*p* < 0.01). Patients in the CS group were more likely to be supported by veno-arterial ECMO compared with those in the LIS group [41 (17%) vs. 9 (9.7%); *p* < 0.01]. Furthermore, there was a significant difference in the INTERMACS profiles between the CS and the LIS groups, with patients in the CS group having worse INTERMACS profiles than the other group (Table 1). A total of 47 patients (19%) in the CS group were on ventilator preoperatively vs. 5 patients (5.4%) in the LIS group (*p* < 0.01). Follow-up was complete in 100% of the patients. There was no big difference in the median follow-up time (until death, censoring for transplant/LVAD removal, or end of follow-up) between the groups [612 (IQR: 160–1,172) days in the CS group vs. 463 (IQR: 204–943) days in the LIS group, *p* = 0.4]. Sixteen LVADs (4.8%) were explanted during the follow-up after recovery of the left ventricular function (2.1% in the LIS and 5.8% in the CS groups).

**Table 1:** Pre- and intraoperative patient characteristics in unmatched and matched cohorts.

Features	Overall cohort			Propensity-matched cohort		
	CS, *N* = 242	LIS, *N* = 93	*p*-Value	CS, *N* = 98	LIS, *N* = 67	*p*-Value
Age (median; IQR)	61 (53–66)	63 (55–66)	0.4	61 (55–66)	64 (58–67)	0.3
Female	32 (13%)	8 (8.6%)	0.2	7 (7.1%)	6 (9%)	0.7
BMI (median; IQR)	28.1 (24.9–31.6)	27.7 (24.0–30.9)	0.6	28.1 (25.2–31.6)	27.9 (24.0–31.1)	0.8
Cardiomyopathy etiology			0.2			0.8
Ischemic	118 (49%)	42 (45%)		46 (47%)	29 (43%)	
Dilatative	113 (47%)	50 (54%)		49 (50%)	38 (57%)	
Others	11 (4.5%)	1 (1.1%)		3 (3.1%)	0 (0%)	
Diabetes mellitus			0.9			0.8
Type I	4 (1.7%)	2 (2.2%)		1 (1.0%)	1 (1.5%)	
Type II	91 (38%)	34 (37%)		44 (45%)	26 (39%)	
AF preoperatively	120 (50%)	45 (48%)	0.8	52 (53%)	34 (51%)	0.8
PAD	33 (14%)	12 (13%)	0.9	17 (17%)	9 (13%)	0.5
Creatinin (µmol/L) (median; IQR)	118 (91–156)	122 (91–165)	0.7	120 (92–164)	127 (98–166)	0.4
eGFR (median, IQR)	55 (39–77)	59 (36–75)	0.7	54 (39–78)	51 (36–69)	0.4
CKD	131 (54%)	46 (49%)	0.4	63 (64%)	37 (55%)	0.2
Dialysis preoperatively	19 (7.9%)	9 (9.7%)	0.6	5 (5.1%)	6 (9.0%)	0.4
Bilirubin (µmol/L) (median, IQR)	15 (10–24)	14 (8–20)	0.2	15 (10–24) *n* = 95	13 (8–17) *n* = 63	0.2
Hemoglobin (mmol/L) (median, IQR)	6.4 (5.7–7.5)	6.8 (5.9–7.8)	0.09	6.7 (6.1–7.7)	6.9 (6.05–7.90)	0.5
COLD	23 (9.5%)	8 (8.6%)	0.8	9 (9.2%)	7 (10%)	0.8
Preoperatively tricuspid valve insufficiency ≥II	100 (43%)	40 (43%)	>0.9	42 (43%)	28 (42%)	0.9
TAPSE (median, IQR)	14.0 (12.0–17.0) *n* = 206	15.0 (12.0–17.0) *n* = 83	0.3	14.0 (12.0–16.0) *n* = 91	15.0 (12.0–16.0) *n* = 66	0.3
History of prior cardiac surgery	67 (28%)	13 (14%)	0.008	21 (21%)	10 (15%)	0.3
Preoperative v-a ECMO	41 (17%)	9 (9.7%)	0.095	12 (12%)	5 (7.5%)	0.3
LVAD type:			0.004			0.9
HVAD	61 (25%)	39 (42%)		26 (27%)	19 (28%)	
HM2	14 (5.8)	1 (1.1%)		–	–	
HM3	167 (69%)	53 (57%)		72 (73%)	48 (72%)	
Concomitant procedures at the time of VAD implantation	69 (29%)	0 (0%)	<0.001	0 (0%)	0 (0%)	>0.9
Hypertension	152 (63%)	55 (59%)	0.5	70 (71%)	42 (63%)	0.2
Intubation preoperatively	47 (19%)	5 (5.4%)	0.001	12 (12%)	5 (7.5%)	0.3
INTERMACS			<0.001			0.3
1/2	114 (47%)	23 (25%)		32 (32%)	16 (24%)	
3/4	67 (28%)	51 (53%)		67 (68%)	51 (76%)	
MAP (mmHg; median; IQR)	77 (70–88) *n* = 177	79 (70–86) *n* = 67	>0.9	80 (71–91) *n* = 73	79 (70–86) *n* = 50	0.3
CI (L/min/m^2^; median; IQR)	1.80 (1.43–2.29) *n* = 177	1.7 (1.4–2.1) *n* = 76	0.3	1.70 (1.40–2.04) *n* = 80	1.73 (1.40–2.10) *n* = 56	0.8
PAP mean (mmHg; median; IQR)	37 (28–43) *n* = 181	35 (28–42) *n* = 75	0.3	37 (30–43) *n* = 81	35 (28–42) *n* = 56	0.3
RVSWI (g*m/m^2^; median; IQR)	6.3 (4.7–8.6)	5.9 (4.6–7.5)	0.4	5.7 (4.5–8.7)	6.2 (4.6–8.0)	0.9
PCWP (median; IQR)	27 (21–32) *n* = 168	24 (18–31) *n* = 68	0.09	28 (21–33) *n* = 75	26 (18–31) *n* = 50	0.12
Off-Pump implantation	1 (0.4%)	11 (12%)	<0.001	1 (1.0%)	0 (0%)	>0.9
CPB time (min, median; IQR)	68 (51–99)	60 (43–80)	0.002	60 (48–77)	63 (49–80)	0.6
Total surgery time (min, median; IQR)	176 (142–236)	195 (169–220)	0.05	164 (135–196)	200 (182–231)	<0.001

AF, atrial fibrillation; BMI, body mass index; CI, cardiac index; CKD, chronic kidney disease; COLD, chronic obstructive lung disease; CPB, cardiopulmonary bypass; CVP, central venous pressure; ECMO, extracorporeal membrane oxygenation, eGFR, estimated glomerular filtration rate; HM2, HeartMate 2, HM3, HeartMate 3, HVAD, HeartWare ventricular assist device; INTERMACS, Interagency Registry for Mechanically Assisted Circulatory Support; IQR, interquartile range; LIS, less-invasive surgery; LVAD, left ventricle assist device; MAP, mean arterial pressure; PAD, peripheral artery disease; PAP, pulmonary arterial pressure; PCWP, pulmonary capillary wedge pressure; RVSWI, right ventricular stroke work index [(meanPAP-CVP)*SI*0.0136]; TAPSE, tricuspid annular plane systolic excursion.

### Outcome

#### Overall population

**Figure 1:**
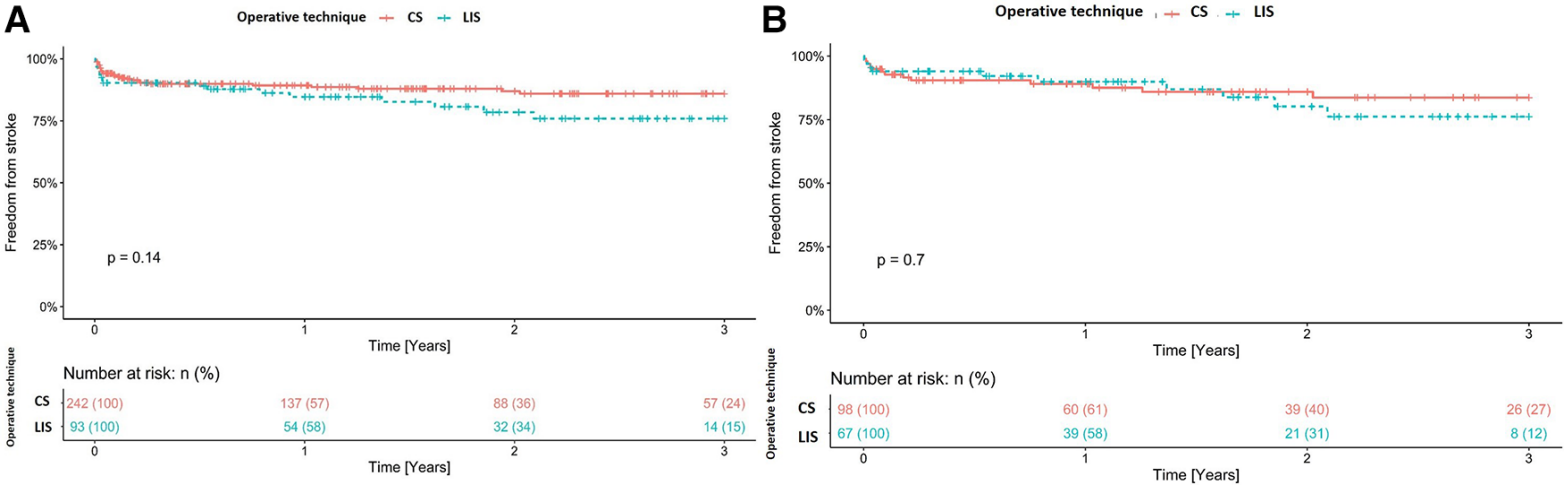
(**A**) Freedom from stroke for the unmatched cohort. (**B**) Freedom from stroke for the matched cohort. CS, conventional sternotomy; LIS, less-invasive surgery.

**Figure 2:**
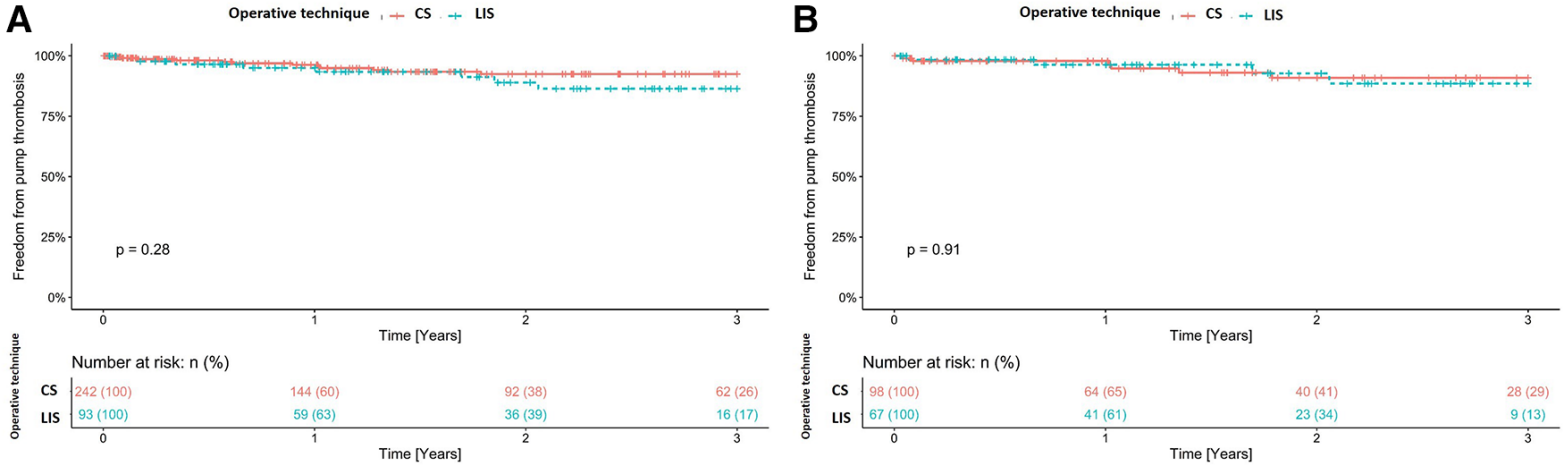
(**A**) Freedom from pump thrombosis for the unmatched cohort. (**B**) Freedom from pump thrombosis for the matched cohort. CS, conventional sternotomy; LIS, less-invasive surgery.

The overall stroke rate, regardless of the implanted device, was 13.7% (46/335). When comparing the groups (CS and LIS) within the cohort, in the unmatched groups, stroke was more frequent in the LIS group (19% vs. 12%, *p* = 0.06) than in the other group (Table 2). Stroke was also more common in patients with the implanted HVAD than in those with HM3 (21% vs. 8.6%; *p* < 0.001). Among HVAD patients, stroke was more common in the LIS group (14.8% vs. 30.8%; *p* = 0.09). In HM3 patients, stroke was more frequent in the CS group (19.4% vs. 11.3%; *p* = 0.6). Table 3 shows the specific distribution of stroke type in the cohort. Stroke after LVAD implantation was associated with significantly higher mortality (*p* > 0.001). Figure 1A shows freedom from stroke for the unmatched cohort. Similarly, prior to matching, pump thrombosis was most common in the LIS group than in the CS group (9.7% vs. 5.8%, *p* = 0.2, Table 2). In the entire cohort pump, thrombosis occurred significantly more often in patients with the HVAD (16%) than in those with HM3 (1.8%; *p* < 0.001; Table 3). Figure 2A shows freedom from stroke for the unmatched cohort. A re-exploration for bleeding was necessary in 45 (19%) patients in the CS group compared with 7 (7.5%) in the LIS group (*p* = 0.01). Furthermore, postoperative dialysis and the number of patients with respiratory insufficiency who needed tracheotomy were significantly more often in the CS group (Table 2). Right heart failure (RHF) after LVAD implantation was more frequent in the CS group than in the LIS group. Postoperative right ventricular assist device (RVAD) use was also significantly higher in the CS group (23% in the CS group vs. 9.7% in the LIS group, *p* < 0.01). Moreover, support time with RVAD was significantly longer for patients in the CS group than in the LIS group [18 (IQR: 9–40) days vs. 10 (IQR: 9–11) days, respectively, *p* = 0.03]. Prior to matching, the concomitant cardiac procedures at the time of LVAD implantation, other than temporary right ventricle support, were performed only in the CS group (*n* = 69, 29%; *p* < 0.001). The most common procedure was aortic valve replacement (*n* = 25), followed by a repair of intracardiac shunts (*n* = 16) and coronary artery bypass graft surgery (*n* = 9). Other valvular procedures (mitral or tricuspid valve repair, *n* = 7) and ascending aorta replacement (*n* = 3) were not commonly performed in our cohort. The duration of ICU stay for the LIS group patients was significantly lower than that for the CS group patients [2 (IQR: 2–4) days vs. 4 (IQR: 2–11) days, respectively, *p* < 0.001]. There was also a significant reduction in the hospital length of stay [36 (IQR: 25–50) days in the CS group vs. 43 (IQR: 27–66) days in the LIS group, *p* = 0.04]. All-cause hospital mortality was significantly higher in the CS group than in the LIS group (17% vs. 6.5%, *p* = 0.01). The most common causes of death were multiorgan failure (25/48; 52.1%), prolonged right heart failure (7/48; 14.6%), hemorrhagic stroke (4/48; 8.3%), ischemic stroke (4/48; 8.3%), and acute respiratory distress syndrome (4/48; 8.3%).

**Table 2:** Postoperative outcomes in conventional surgery and less-invasive surgery groups in unmatched and matched cohorts.

Outcome	Overall cohort			Propensity-matched cohort		
	CS, *n* = 242	LIS, *n* = 93	*p*-Value	CS, *n* = 98	LIS, *n* = 67	*p*-Value
Re-exploration for bleeding	45 (19%)	7 (7.5%)	0.01	18 (18%)	6 (9.0%)	0.09
**Blood products (median; IQR)**						
RBC	9 (3–19) *n*: 158	5 (3–8) *n*: 67	0.012	6 (3–13) *n*: 67	6 (3–8) *n*: 48	0.5
Plasma	5 (2–10) *n*: 152	3 (0–6) *n*: 63	0.027	4 (0–8) *n*: 65	4 (0–6) *n*: 45	0.6
Thrombocytes	2.0 (0–5) *n*: 157	2.0 (0–2) *n*: 66	0.016	2 (0–3) *n*: 66	2 (0–2) *n*: 68	0.3
RVAD implantation	56 (23%)	9 (9.7%)	<0.01	19 (19%)	7 (10%)	0.12
RVAD support duration days (median, IQR)	18 (9–40)	10 (9–11)	0.03	24 (12–56)	10 (10–12)	0.03
Stroke	28 (12%)	18 (19%)	0.06	13 (13%)	11 (16%)	0.6
Ischemic	17	12		10	9	
Hemorrhagic	11	6		3	2	
Pump thrombosis	14 (5.8%)	9 (9.7%)	0.2	6 (6.1%)	5 (7.5%)	0.8
Dialysis			0.01			0.06
Acute	63 (26%)	19 (20%)		21 (21%)	14 (21%)	
Chronic	34 (14%)	4 (4.3%)		16 (16%)	3 (4.5%)	
Tracheotomy	70 (29%)	14 (15%)	<0.01	29 (30%)	11 (16%)	0.04
Driveline infection	76 (31%)	25 (27%)	0.4	31 (32%)	20 (30%)	0.8
Wound infection	22 (9.1%)	3 (3.2%)	0.07	7 (7.1%)	2 (3.0%)	0.3
GIB	24 (9.9%)	9 (9.7%)	>0.9	8 (8.2%)	5 (7.5%)	0.9
Sepsis	26 (11%)	6 (6.5%)	0.2	14 (14%)	5 (7.5%)	0.2
Heart transplantation during follow-up	41 (17%)	19 (20%)	0.5	15 (15%)	14 (21%)	0.4
Follow-up days (median; IQR)	542 (133–1,118)	518 (236–995)	0.6	612 (160–1,172)	463 (204–943)	0.4
ICU stay days (median; IQR)	4 (2–11)	2 (2–4)	<0.001	4 (2–12)	2 (2–5)	<0.01
Hospital length of stay (median; IQR)	43 (27–66)	36 (25–50)	0.04	38 (26–55)	36 (25–55)	0.6
30-day mortality	21 (8.7%)	5 (5.4%)	0.3	5 (5.1%)	5 (7.5%)	0.5
Hospital mortality	42 (17%)	6 (6.5%)	0.01	19 (19%)	5 (7.5%)	0.03
1-year mortality	66 (27.3%)	15 (16.1%)	0.035	24 (24.5%)	12 (17.9%)	0.35

CS, conventional sternotomy; CPB, cardiopulmonary bypass; GIB, gastrointestinal bleeding; ICU, intensive care unit, IQR, interquartile range; LIS, less-invasive surgery; RBC, red blood cells; RHF, right heart failure; RVAD, right ventricular assist device.

**Table 3:** Distribution of stroke and pump thrombosis in the unmatched cohort regard to implanted devices.

	Unmatched cohort (*n* = 335)					
	HVAD (*n* = 100)			HM 3 (*n* = 220)		
	CS (*n* = 61)	LIS (*n* = 39)	*p*-Value	CS (*n* = 167)	LIS (*n* = 53)	*p*-Value
Stroke all	9 (14.8%)	12 (30.8%)	0.09569	13 (19.4%)	6 (11.3%)	0.6045
Stroke ischemic	6	7	0.3833	9	5	0.5793
Stroke hemorrhagic	3	5	0.297	3	1	1
Pump thrombosis	8 (13.1%)	8 (20.5%)	0.481	3 (1.8%)	1 (1.9%)	1

CS, conventional sternotomy; HM3, HeartMate 3; HVAD, HeartWare ventricular assist device; LIS, less-invasive surgery.

The cumulative mortality rate at 1 year was also significantly higher in the CS group (27.3%). In the univariable analysis, the type of the LVAD used and off-pump LVAD implantation were identified as risk factors for stroke. Concomitant occlusion of the left atrial appendage has a preventive effect (Supplementary Appendix). Similarly, the type of LVAD has been identified as an influencing factor for the occurrence of pump thrombosis (Supplementary Appendix).

### Propensity-matched cohort

In total, 67 patients in the LIS group were matched with 98 patients in the CS group. The variables used in the propensity score match were mentioned previously (statistical methods). After the groups were matched, no significant difference in postoperative severe acute RHF requiring RVAD implantation was observed (CS 19% vs. LIS 10%, *p* = 0.12). However, the RVAD support time was significantly shorter in the LIS group [10 (IQR: 10–12) days in the LIS group vs. 24 (IQR: 12–56) days in the CS group; *p* = 0.03]. Moreover, in the matched cohort, 53% patients with mild RVF, 15% with moderate RVF, 11% with severe RVF, and 20% with severe acute RVF were identified in the CS group. In contrast, in the LIS group, 69% patients with mild RVF, 7.5% with moderate RVF, 10% with severe RVF, and 12% with severe acute RVF were identified. In the statistical analysis, no significant different was observed.

The need to perform a re-exploration because of bleeding was less in the LIS group (9% vs. 18%, *p* = 0.09). LIS was also associated with a shorter ventilation time and a significantly lower rate of tracheotomy (16% vs. 30%, *p* = 0.04). The duration of ICU stay for the LIS group was significantly shorter than that for the CS group [2 (IQR: 2–5) days vs. 4 (IQR: 2–12) days, *p* < 0.01]. However, there was no significant reduction in the hospital length of stay [36 (IQR: 25–55) days vs. 38 (IQR: 26–55) days, *p* = 0.6]. The overall stroke rate in the matched cohort was 14.5% (*n* = 24). There was no significant difference in the incidence rate of stroke between both groups [14% (13/98) in the CS group vs. 16% (11/67) in the LIS group; *p* = 0.6; Table 2]. Perioperative stroke (within 30 days of surgery) occurred in 5 (5.1%) patients in the CS group and in 4 (6.0%) in the LIS group. In the first 6 months after LVAD implantation, we observed collectively 9 (9.2%) stroke events in the CS group and 4 (6.0%) in the LIS group. Figure 1B shows freedom from stroke for the matched cohort. The incidence rate of pump thrombosis during follow-up was also not significantly different between the groups [6.1% (6/98) in the CS group vs. 7.5% (5/67) in the LIS group; *p* = 0.8]. Figure 2B shows freedom from stroke for the matched cohort. In the matching cohort, no concomitant procedure was performed in both groups of patients. To better investigate the impact of the pump type on the outcome and after excluding HVAD and HM II patients, a total of 123 patients were identified in the matched cohort (*n* = 74 in the CS group and *n* = 49 in the LIS group). In this subanalysis, there was no statistical difference in the overall mortality rate during follow-up (41% in the CS group vs. 29% in the LIS group, *p* = 0.2); in the stroke rate (8.1% vs. 10%; *p* = 0.8), and in the pump thrombosis rate (2.7% vs. 2.0%, *p* > 0.9).

**Figure 3:**
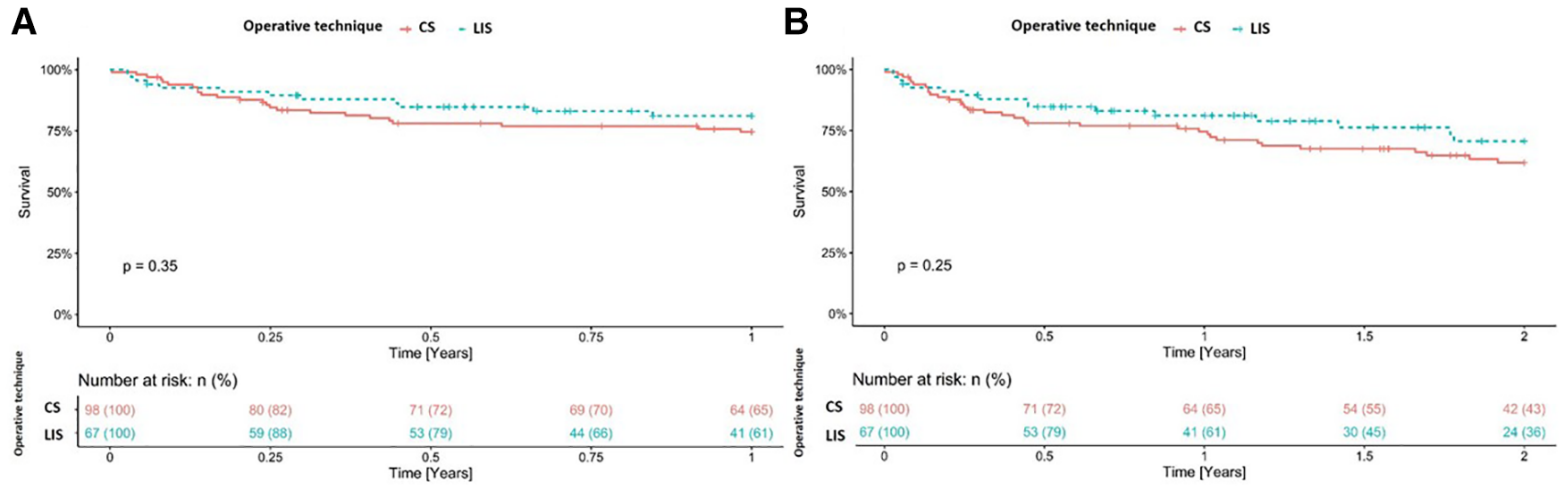
Kaplan–Meier curve for all-cause mortality at 1 year (**A**) and 2 years (**B**) in the propensity-matched cohort. CS, conventional sternotomy; LIS, less-invasive surgery.

The hospital mortality rate was significantly lower in the LIS group (7.5% vs. 19%; *p* = 0.03). However, the 1-year mortality showed no significant difference 24.5% in the CS group vs. 17.9% in the LIS group (*p* = 0.35) (Figures 3A,B). Postoperative incidence of driveline infection, wound infection, gastrointestinal bleeding, and the need for hemodialysis were not significantly different between the groups during follow-up. Similarly, the rate of heart transplantation with the device did not differ between both groups.

## Discussion

The outcomes of patients who received LVAD implantation have significantly improved over the last 10 years. The main reasons for this are advances in device design, better patient selection, and improved postoperative management (8). Nevertheless, stroke remains a significant complication after LVAD placement. It is a leading cause of death that affects not only outcomes but also the quality of life and transplantation candidacy (9–11). The overall stroke rate in our cohort, regardless of the implanted device, was 13.7%, which is similar to that in previous published results (12–14). The analysis of the matched cohort showed no differences in the stroke incidence rate between the groups (CS 13% vs. LIS 16%, *p* = 0.6; Table 2). There are no large studies that have investigated the stroke rate and its correlation with the surgical approach. In the MOMENTUM 3 trial, the HM3 device was associated with almost half the risk of stroke compared with the HM2 device at 2 years of follow-up (10.1% vs. 19.2%; *p* = 0.02) (13). In contrast, data from the HeartWare HVAD pivotal trials showed an increased risk of stroke compared with the HM2 device. In the ENDURANCE DT trial, a significantly higher number of HVAD patients compared with HM2 patients experienced a stroke at 2 years (29.7% vs. 12.1%; *p* < 0.001) (14). Our results are similar to those of Chiang and colleagues (15). They conducted an unmatched single-center study of 247 total patients comparing HVAD (*n* = 163) vs. HM3 (*n* = 84) with regard to stroke during a median follow-up of 1.2 years in HVAD patients and 1.4 years in HM3 patients. In this context, it is important to mention that patients under ECMO support were excluded from this analysis. Their results showed an overall stroke rate of 12.2% (30/247). Stroke occurred in 24 (14.7%) HVAD patients (15 ischemic and 9 hemorrhagic) and 6 (7.1%) HM3 patients (4 ischemic and 2 hemorrhagic). In multivariate analysis, the HVAD was found to be associated with a significantly higher stroke risk (HR, 2.57; 95% confidence interval, 1.02–6.44; *p* = 0.045). In our unmatched cohort, we observed upon multivariate analysis, almost more than twice the number of stroke events in HVAD patients than in HM3 ones (hazard ratio, 3.31; *p* = 0.017). In the matched cohort, no statistical significance was seen because of the low number of events in HM3 patients (HR, 3.05; *p* = 0.175). There was no significant difference between the devices and surgical technique used in the unmatched cohort (Table 3). The LATERAL study was a multicenter, prospective, and nonrandomized trial that evaluated the lateral thoracotomy implantation of the HVAD and compared these results with previous historical data from the sternotomy approach (2). A total of 12 out of 144 (8.4%) subjects were reported to have had a stroke within 6 months postimplant, which was evaluated by using the modified Rankin Scale. These results are comparable to our observation. In our study, the stroke rate in the matched cohort within 6 months postimplant was 7.9% (13/165 for both the CS and LIS groups). The overall stroke rate for the LIS group was 21% in HVAD patients and 12.5% in HM3 patients. In our study, we included all postoperative stroke events (hemorrhagic and ischemic), which were validated by computed tomography; also incidental findings were reported without clinical correlation. This and the longer follow-up could explain the higher rate of stroke in HVAD patients. The LIS approach is technically more demanding. Consequent on such technical difficulties are usually longer operative times. However, in our matched cohort, we observed no difference in CPB time (*p* = 0.6). The detrimental effects of CPB are already well known. These include systemic immune inflammatory response with platelet damage and fibrinolysis, which cause renal dysfunction, acute lung injury, and stroke (16, 17). Moreover, platelet dysfunction and coagulopathy that occur after a CPB increase the risk of perioperative bleeding and the need for blood transfusion, which, in turn, contribute to volume overload and possible RHF. Alternatively, the less-invasive implantation can be performed without using the CPB machine. However, the off-pump approach is associated with a limited exposure of the left ventricular space and can potentially increase the risk of pump thrombosis because thrombi in the left ventricle may not be detected and removed. Hospital mortality was significantly higher in the CS group in the unmatched cohort. The overall mortality was still higher in the CS group but did not reach statistical significance. Following propensity score matching, the hospital mortality rate was significantly lower in the LIS group. This finding merits careful observation in future studies. Our results support the efforts of previous studies in investigating the use of the less-invasive LVAD implantation technique (2, 3, 18, 19). Another important observation in this study was the fact that even after the groups were matched, the LIS group was associated with a lower tracheotomy rate (16% vs. 30%, *p* = 0.05). This difference may be explained by the limited occurrence of surgical trauma and faster recovery for patients with LIS. This is very well mirrored by the shorter ICU stay in the LIS group (2 vs. 4 days, *p* < 0.01). Moreover, the full-sternotomy sparing operation is associated with a better postoperative stability of the thorax and thereby supports the respiratory function and faster weaning from mechanical ventilation. In this study, we observed a decreased incidence of severe RV failure when utilizing an LIS approach for LVAD implantation. This finding has now been well documented across several studies (4, 5, 20). There are many theories of possible protective effects of the LIS approach. Studies have indicated that pericardial opening promotes RV dilatation and changes in the pressure–volume relationship, resulting in impaired RV function (21). Therefore, the preservation of the pericardial restraint over the RV is crucial during the performance of the operation. Moreover, the minimal heart displacement during the LIS approach avoids potential coronary hypoperfusion and preserves the septal function. It has been shown that the septal function constitutes the highest share of the total RV function (22). In our cohort, in the LIS group, only limited pericardial opening was performed with additional closing of the pericardium directly or by the use of a membrane after LVAD implantation. In addition, we found that LIS was associated with a lesser need for postoperative re-exploration for bleeding. Moreover, the LIS patients demonstrated less blood product utilization including fewer packed red blood cells (*p* = 0.012), less plasma (*p* = 0.027), and fewer platelets (*p* = 0.016). Our results are similar to the previous findings (2–5). It is important to note that the avoidance of reoperation and less blood transfusion have a protective effect on the right ventricle function.

### Limitations

Several limitations of this study merit consideration. The main limitation of this study is its retrospective nature and the fact that the patients were not randomized to a surgical approach. Therefore, the patient groups were not identical. To achieve a high level of similarity in preoperative characteristics, we used both multivariable analysis regression and propensity matching. As a result, unlike randomized control trials, propensity score analyses have the limitation that some unmeasured confounding variables may still be present, thus leading to biased results. Because patients with a low INTERMACS of 1 or 2 (114/137 in CS vs. 23/137 in LIS, *p* = 0.00019) and a higher CRP (22 in the CS group vs. 8.05 in the LIS group, *p* = 0.00036) were more likely to receive total sternotomy, these patients were partially excluded by propensity matching as part of the preoperative comparability of patients. Therefore, our results in the matched cohorts have limited applicability to these patients. It should also be noted that in the years up to 2018, LVAD was performed more frequently by using CS rather than LIS, whereas in the last 3 years, the minimally invasive method was preferred. Also the observation periods of the individual patients postoperatively, depending on the time of implantation, can vary greatly and range from a few months to 6 years. However, we can exclude additional uncontrolled factors, which could influence survival and adverse events. Notably, two experienced surgeons performed the LVAD implantation, and this study was limited to a single institution. Our center usually performs a high volume of surgeries for heart failure. Nevertheless, while two different surgeons performed both procedures, this study remains limited to a single institution and may not cover other centers.

## Conclusion

In summary, a less-invasive strategy for LVAD implantation is a good alternative procedure to conventional LVAD implantation by full median sternotomy with a potential advantage in the early postoperative period. Postoperative stroke and pump thrombosis remain comparable to the sternotomy approach. Further, no significant difference in all-cause mortality during follow-up was observed. A randomized controlled trial comparing the CS and LIS approaches may be necessary to confirm our findings.

## Data Availability

The raw data supporting the conclusions of this article will be made available by the authors without undue reservation.
