# Supplementary material for: Stroke and pump thrombosis following left ventricular assist device implantation: The impact of the implantation technique
Source: Front Cardiovasc Med. 2023 May 11;10:974527. doi: 10.3389/fcvm.2023.974527 (PMC10213891; doi:10.3389/fcvm.2023.974527)
Supplement: Supplementary file 1 [file Table1.docx]

Pump thrombosis is an event in which the pump or its conduits contain a thrombus that results in or could potentially induce circulatory failure.

Suspected pump thrombus is an event in which clinical or mechanical circulatory support device parameters suggest thrombus in the blood contacting components of the pump, cannulae, or grafts. Signs and symptoms should include at least 2 of the 3 following criteria:

1. Presence of hemolysis (clinical hemolysis and/or sustained LDH >3.0 upper laboratory normal limit)

2. Worsening heart failure (or lack of left ventricular unloading when a ramp test is performed)

3. Abnormal pump parameters (elevated pump powers >10 W or 2 W higher than baseline)

Suspected pump thrombus should be accompanied by ≥1 of the following events or interventions:

1. Treatment with intravenous anti-coagulation (e.g., heparin), intravenous thrombolytics (e.g., tPA), or intravenous anti-platelet therapy (e.g., eptifibatide, tirofiban)

2. Pump replacement

3. Pump explantation

4. Urgent transplantation by recurrent pump thrombosis

5. Stroke

6. Death

|  | Overall cohort N=335 (all devices) | | |
| --- | --- | --- | --- |
| Features | No stroke N= 289 | Stroke N= 46 | P-Value |
| LVAD-Type:  HVAD  HM2 HM3 | 79 (27%)  9 (3.0%)  201 (70%) | 21 (46%)  6 (13%)  19 (41%) | < 0.001 |
| Concomitant LAA occlusion | 32 (11%) | 1 (2.2%) | 0.06 |
| Off-Pump implantation | 6 (2.1%) | 6 (13%) | 0.002 |

**Table 4. Univariate analysis of risk factors for stroke.**

**HM2: HeartMate2; HM3: HeartMate 3; HVAD: HeartWare ventricular assist device; LVAD: left ventricle assist device**

|  | Overall cohort N=335 (all devices) | | |
| --- | --- | --- | --- |
| Features | No pump thrombosis  N= 312 | Pump thrombosis  N= 23 | P-Value |
| Age (median; IQR) | 61 (54-67) | 56 (48-63) | 0.045 |
| LVAD-Type:  HVAD  HM2 HM3 | 84 (27%)  12 (3.8%)  216 (69%) | 16 (70%)  3 (13%)  4 (17%) | < 0.001 |

**Table 5. Univariate analysis of risk factors for pump thrombosis.**

**HM2: HeartMate2; HM3: HeartMate 3; HVAD: HeartWare ventricular assist device; LVAD: left ventricle assist device**
